# Correction to: Epigenetic signatures associated with imprinted paternally expressed genes in the *Arabidopsis* endosperm

**DOI:** 10.1186/s13059-019-1803-3

**Published:** 2019-09-02

**Authors:** Jordi Moreno-Romero, Gerardo Del Toro-De León, Vikash Kumar Yadav, Juan Santos-González, Claudia Köhler

**Affiliations:** 1 Present Address: Centre for Research in Agricultural Genomics (CRAG), CSIC-IRTA-UAB-UB, Campus UAB, Bellaterra, Barcelona, Spain; 2 Department of Plant Biology, Uppsala BioCenter, Swedish University of Agricultural Sciences and Linnean Center for Plant Biology, Uppsala, Sweden

**Correction to: Genome Biol**

**https://doi.org/10.1186/s13059-019-1652-0**

Following publication of the original article [1], the authors reported that Additional file 4, “Table S5. Parent-of-origin RNAseq dataset of 4 DAP INTACT-purified endosperm of Col × L*er* reciprocal crosses” had the following error:

- Column 6, labeled as “Reads_Mat_Ler”, should say “Reads_pat_Col”.
- Column 7, labeled as “Reads_pat_Ler”, should say “Reads_mat_Ler”.

The updated Additional file 4 is published in this correction.

## Additional file

Additional file 4: **Table S5.** Parent-of-origin RNAseq dataset of 4 DAP INTACT-purified endosperm of Col × L*er* reciprocal crosses. (XLSX 820 kb)
