# Supplementary material for: Epigenetic signatures associated with imprinted paternally expressed genes in the Arabidopsis endosperm
Source: Genome Biol. 2019 Feb 21;20:41. doi: 10.1186/s13059-019-1652-0 (PMC6385439; doi:10.1186/s13059-019-1652-0)
Supplement: Supplementary file 1 — Figure S1. H3K27me3, H3K9me2, and CHG methylation overlap on maternal alleles of PEGs in crosses Ler × Col. Figure S2. Total and parental-specific H3K27me3, H3K9me2, and CHG methylation levels in the endosperm of previously described PEGs. Figure S3. Increasing levels of H3K27me3 on maternal alleles correlates with increasing levels of H3K9me2. Figure S4. Maternal alleles remain silenced in suvh4,5,6 and cmt3 mutants. Figure S5. Paternally biased expression coincides with the combination of H3K27me3, H3K9me2, and CHG methylation in crosses Ler × Col (for reciprocal cross direction see Fig. 3). Table S3. GO terms enrichment of genes in the highest score category. Table S4. Fluorescence analysis of 4 DAP seeds after reciprocal crosses. Table S6. List of primers used in this study. Table S7. Quality of sequencing samples. (PDF 1499 kb) [file 13059_2019_1652_MOESM1_ESM.pdf]

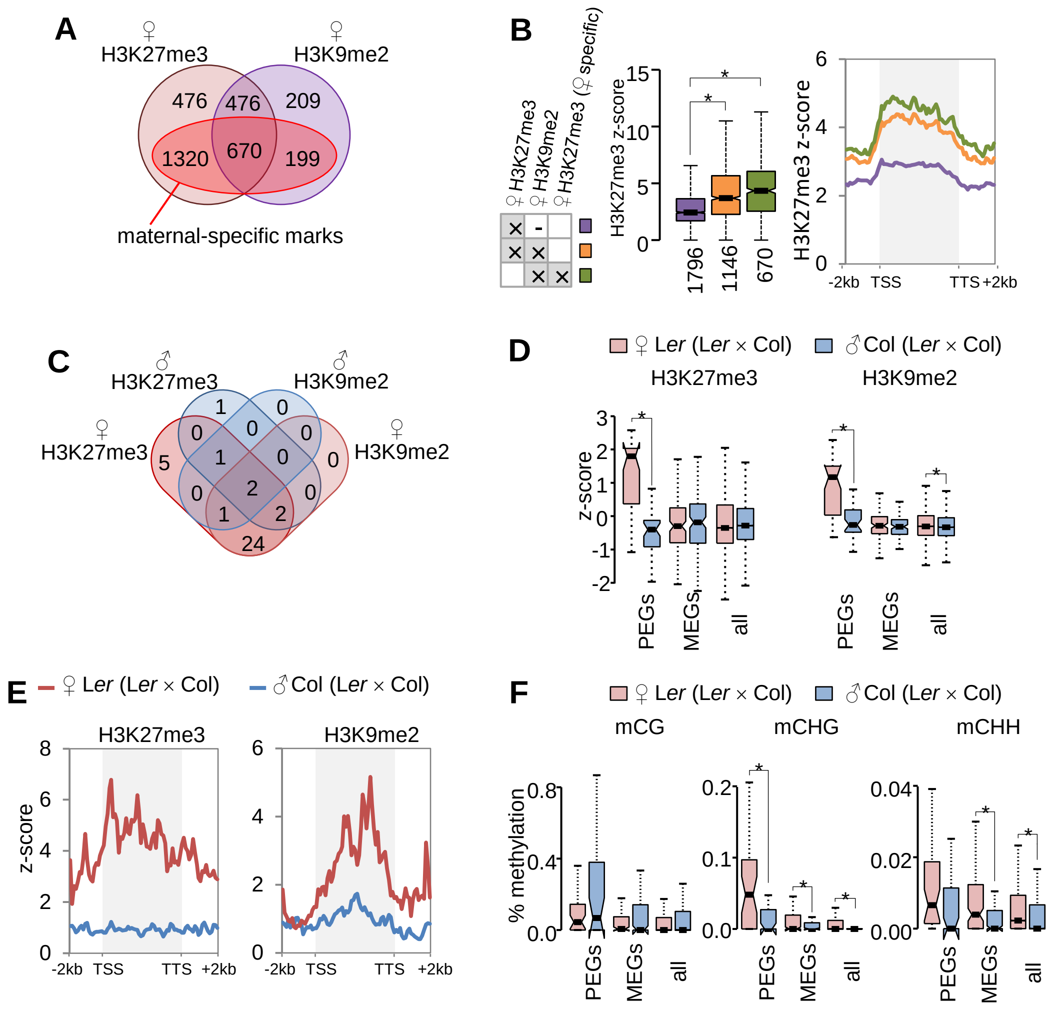


**Fig. S1**

H3K27me3, H3K9me2, and CHG methylation overlap on maternal alleles of PEGs in crosses *Ler* × Col.

**a** Overlap of genes marked by H3K27me3 and H3K9me2 on maternal alleles. Dark red inset marks genes that have H3K27me3 and H3K9me2 exclusively on their maternal alleles (excluding genes also marked on the paternal alleles).
**b** Genes marked by H3K27me3 and H3K9me2 have higher levels of H3K27me3. Box plots show mean values of z-scored H3K27me3 and H3K9me2 of genes where the maternal alleles are either marked by H3K27me3 alone or by a combination of H3K27me3 and H3K9me2. Genes containing maternal-specific H3K27me3 and H3K9me2 are particularly enriched for H3K27me3. Boxes show medians and the interquartile range, error bars show the full range excluding outliers. Notches show the 95% confidence interval for the median. Asterisks mark significance (Wilcoxon rank sum test, *P*-value <5.0E-3).
**c** The majority of PEGs are marked by H3K27me3 and H3K9me2 on their maternal alleles. Overlap of PEGs (Pignatta et al., 2014) with maternal and paternal H3K27me3 and H3K9me2.
**d** Levels of H3K27me3 and H3K9me2 on PEGs, MEGs and all genes. Box plots (as specified in panel b) represent mean values of z-scored H3K27me3 and, H3K9me2 Asterisks mark significance (Wilcoxon rank sum test, *P*-value <5.0E-3).
**e** Metagene plots showing average distribution of z-score normalized H3K27me3 and H3K9me2 levels on maternal and paternal alleles of PEGs (Pignatta et al., 2014).
**f** Levels of DNA methylation in each sequence context (mCG, mCHG and mCHH) on PEGs, MEGs, and all genes. Box plots (as specified in panel b) show mean values of relative DNA methylation. Asterisks mark significance (Wilcoxon rank sum test, *P*-value <5.0E-2).
Data shown correspond to the cross *Ler* x Col, for reciprocal cross direction see Fig. 1.

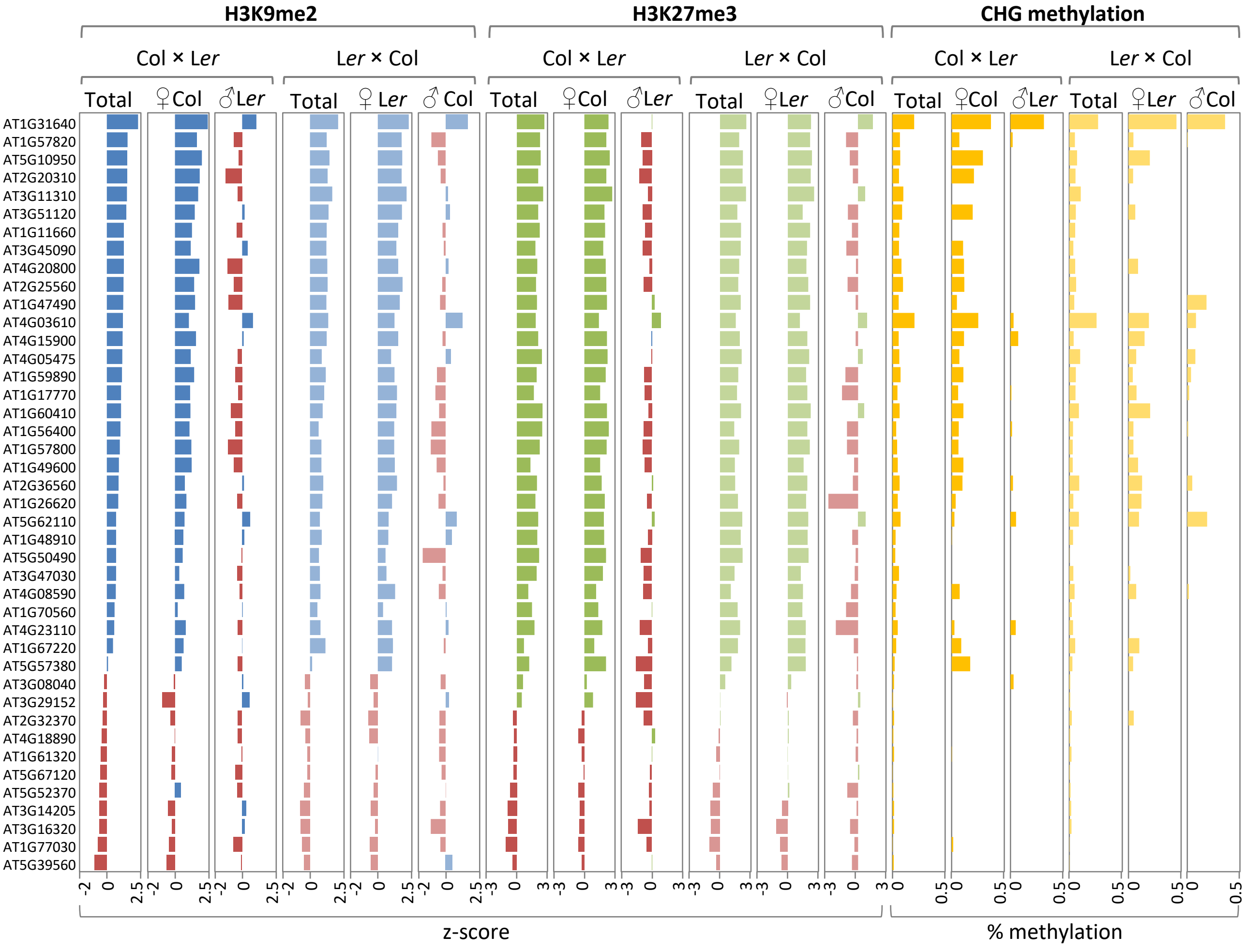


**Fig.S2**

Total and parental-specific H3K27me3, H3K9me2, and CHG methylation levels in the endosperm of previously described PEGs [14].

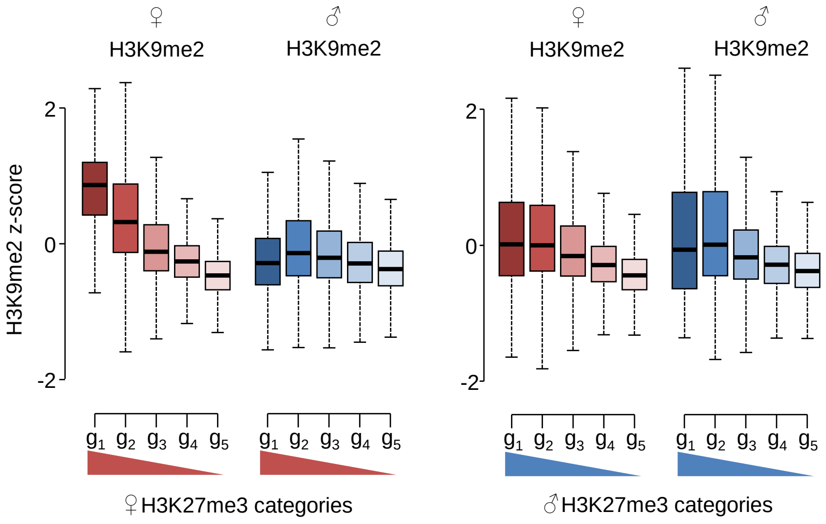


**Fig. S3**

Increasing levels of H3K27me3 on maternal alleles correlates with increasing levels of H3K9me2.

Levels of maternal and paternal H3K9me2 on genes that have been grouped based on their level of H3K27me3 on maternal (left panels) and paternal alleles (right panels) from highest ($g_1$) to lowest ($g_5$) levels. For the z-score rank and number of genes in each category see Additional file 4: Table S5.

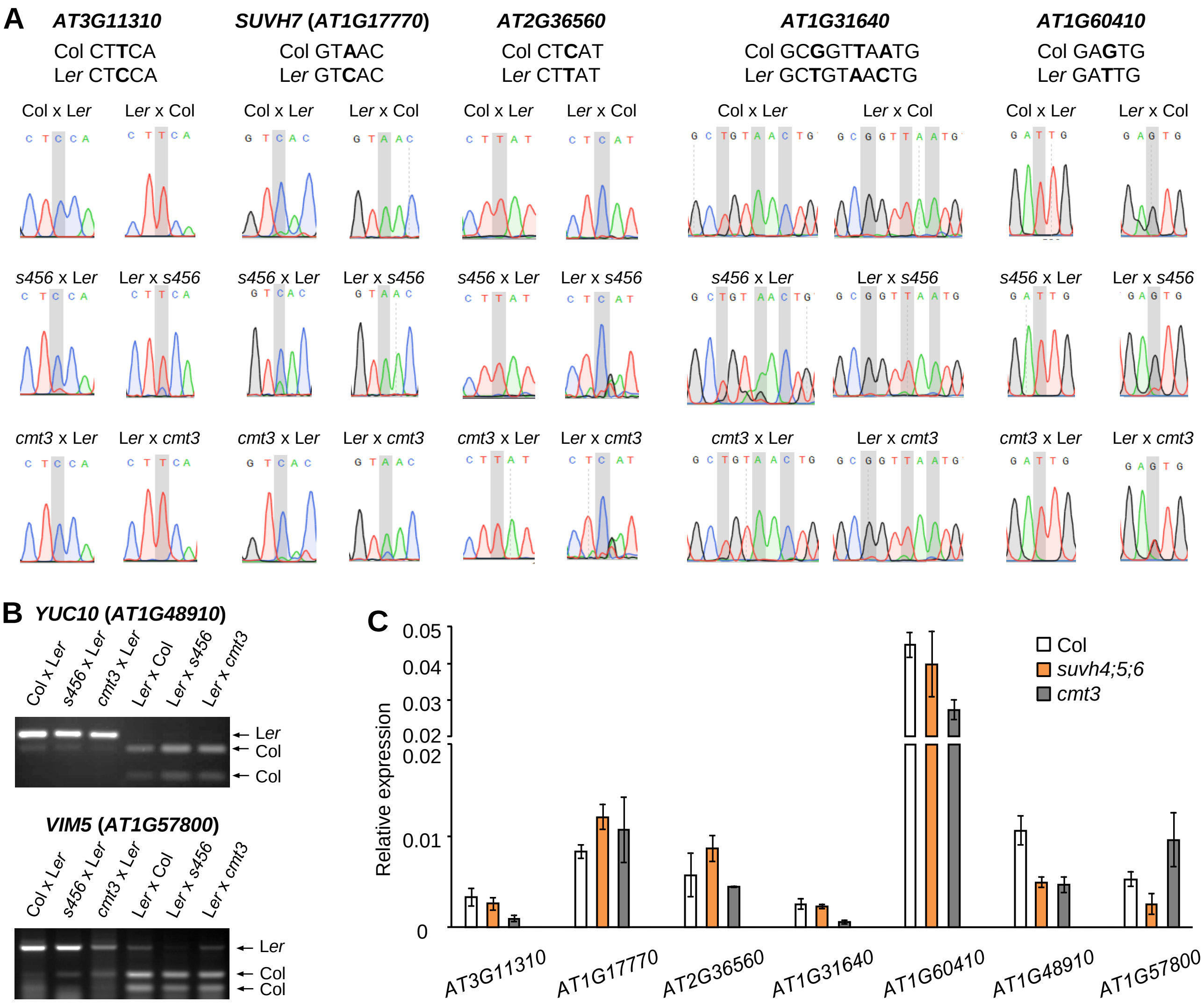


**Fig. S4**

Maternal alleles remain silenced in *suvh4,5,6* and *cmt3* mutants.

**a** Imprinting assay of indicated PEGs in reciprocal crosses of Col and L*er* and mutants for *suvh4,5,6* (*s456*) and *cmt3* using Sanger -sequencing. Nucleotide polymorphisms allowing to distinguish parental alleles are shaded.
**b** Imprinting assay of indicated PEGs in reciprocal crosses of Col and Ler and mutants for *suvh4,5,6* and *cmt3* using restriction-based analysis.
**c** qRT-PCR analysis of indicated PEGs in seeds of Col wild-type, *cmt3* and *suvh4,5,6* mutants at 4 days after pollination. *PP2A* was used as a reference gene. Error bars show SEM. Expression changes in mutants are not significantly different compared to wild type (two-tailed t-test, $P<0.05$).

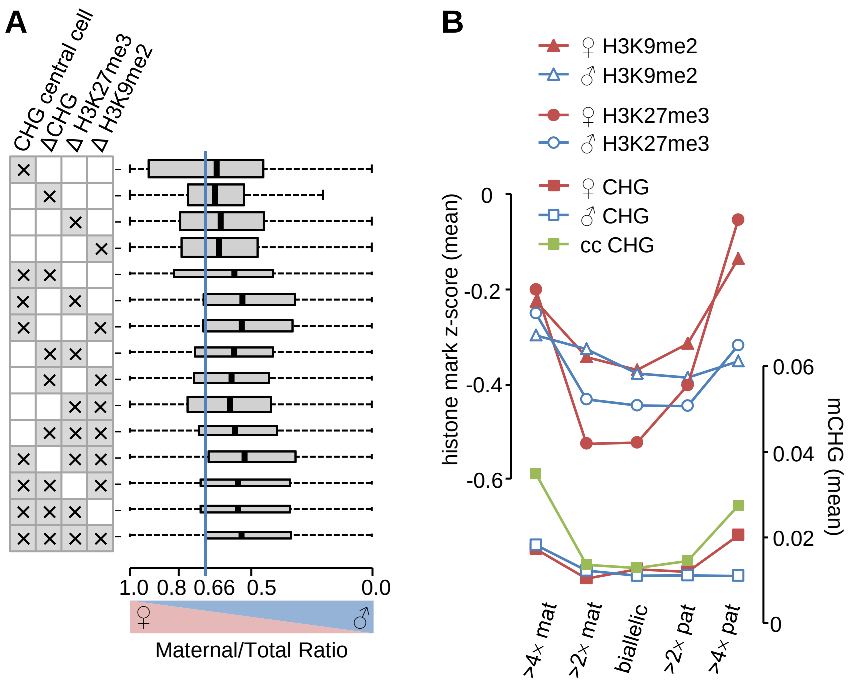


**Fig. S5**

Paternally-biased expression coincides with the combination of H3K27me3, H3K9me2, and CHG methylation in crosses L*er* × Col (for reciprocal cross direction see Fig. 3).

**a** Box plots show mean values of maternal to total reads for genes marked by one or several of the following modifications: CHG methylation in the central cell, CHG methylation, H3K27me3, or H3K9me2 on the maternal alleles in the endosperm. For each modification only genes with top scores were included in the analysis (see Additional File 2: Table S1). Boxes show medians and the interquartile range, error bars show the full range excluding outliers. Widths of boxes are proportional to square-root of the number of genes in each category. Blue line indicates the expected ratio for biallelically expressed genes.

**b** Paternal expression bias correlates with high levels of CHG methylation in the central cell and H3K27me3 and H3K9me2 on maternal alleles in the endosperm. Plotted are mean values of CHG methylation, H3K27me3, and H3K9me2 on maternal and paternal alleles for genes with deviating parental expression levels from the expected two maternal to one paternal ratio.

## Table S3.

GO terms enrichment of genes (P-value <0.01) in the highest score category (Score 12, see Additional File 2: Table S1).

| GO term | P-value | No. of genes | Description |
|---|---|---|---|
| **biological process** | | | |
| GO:0006350 | 7.54E-05 | 28 | transcription |
| GO:0045449 | 7.62E-05 | 27 | regulation of transcription |
| GO:0019219 | 8.91E-05 | 27 | regulation of nucleobase, nucleoside, nucleotide and nucleic acid metabolism |
| GO:0006351 | 1.36E-04 | 19 | transcription, DNA-dependent |
| GO:0031323 | 1.47E-04 | 27 | regulation of cellular metabolism |
| GO:0019222 | 1.89E-04 | 27 | regulation of metabolism |
| GO:0006355 | 2.64E-04 | 18 | regulation of transcription, DNA-dependent |
| GO:0051244 | 7.57E-04 | 27 | regulation of cellular physiological process |
| GO:0050794 | 8.14E-04 | 27 | regulation of cellular process |
| GO:0050791 | 1.12E-03 | 27 | regulation of physiological process |
| GO:0050789 | 1.14E-03 | 29 | regulation of biological process |
| GO:0016568 | 1.57E-03 | 4 | chromatin modification |
| GO:0006139 | 2.30E-03 | 34 | nucleobase, nucleoside, nucleotide and nucleic acid metabolism |
| **molecular function** | | | |
| GO:0030528 | 3.85E-04 | 28 | transcription regulator activity |
| GO:0016782 | 7.63E-04 | 3 | transferase activity, transferring sulfur-containing groups |
| GO:0003677 | 2.45E-03 | 29 | DNA binding |
| GO:0003700 | 4.42E-03 | 23 | transcription factor activity |
| GO:0003676 | 5.91E-03 | 35 | nucleic acid binding |
| GO:0004722 | 6.20E-03 | 5 | protein serine/threonine phosphatase activity |
| GO:0008415 | 8.24E-03 | 5 | acyltransferase activity |
| GO:0016747 | 8.66E-03 | 5 | transferase activity, transferring groups other than amino-acyl groups |
| **cellular component** | | | |
| GO:0005634 | 2.53E-03 | 30 | nucleus |

**Table S4.**

Fluorescence analysis of 4DAP seeds derived after indicated reciprocal crosses.

| **Crosses** | **Line** | **No. of seeds observed** | **Expression in seed coat** | **Expression in endosperm** |
|---|---|---|---|---|
| *AT1G47530-GFP* × WT | L1 | 63 | 60 | 0 |
| | L2 | 58 | 58 | 0 |
| | L3 | 52 | 52 | 0 |
| WT × *AT1G47530-GFP* | L1 | 62 | 0 | 0 |
| | L2 | 52 | 0 | 0 |
| | L3 | 56 | 0 | 0 |
| *AT1G64660-GFP* × WT | L1 | 52 | 52 | 0 |
| | L2 | 53 | 53 | 0 |
| | L3 | 55 | 55 | 0 |
| WT × *AT1G64660-GFP* | L1 | 62 | 0 | 53 |
| | L2 | 53 | 0 | 45 |
| | L3 | 57 | No one | 48 |

## Table S6.

List of primers used in this study.

### Imprinting-by-sequencing

| gene | | Sequence 5' – 3' | product size (bp) |
|---|---|---|---|
| AT1G31640 | FW | ACCAGTAGGGACATAGGGCTG | (Col/Ler) 334/334 |
| | RV | TCTTAATCAGCTTAATGCCAGG | |
| AT1G60410 | FW | TCGTTGGAGTAATCTCTGGTCG | (Col/Ler) 808/808 |
| | RV | TCCCACCTTGGAGTAGTCATGG | |
| AT3G11310 | FW | TGTAGAACCTGGAAAGACCGCTT | (Col/Ler) 385/385 |
| | RV | CGGAGTAGGCTCATGATTGTCATTA | |
| AT1G17770 | FW | GTGAAATGTGCTTAGTGGGGCTT | (Col/Ler) 271/271 |
| | RV | CCTCTCACGACTCTAACGTCATTCC | |
| AT2G36560 | FW | GAACGATGAGTCCAACGATGTGA | (Col/Ler) 363/365 |
| | RV | GGGTTAAACTCATCGTTCATCTCCT | |

### Imprinting-by-restriction enzyme digestion

| gene | | Sequence 5' – 3' | product size (bp) | digestion fragments |
|---|---|---|---|---|
| AT1G57800 (VIM5) | FW | AAAGACCTTGGTTCCTAACAGG | (Col/Ler) 164/164 | digestion BmsI (Col/Ler) 71+93/164 |
| | RV | CTATGTTGACAATGAAGATCGTCC | | |
| AT1G48910 (YUC10) | FW | GTTATCAATGGTGGGATTGGAA | (Col/Ler) 289/289 | digestion HpaII (Col/Ler) 212+53+24/212+77 |
| | RV | CCAAGATAGATCTGATGTCGTC | | |

### Expression analysis

| gene | | Sequence 5' – 3' |
|---|---|---|
| AT1G31640 | FW | GACAACTCTCTGTGACATCAAAGCAT |
| | RV | CGAAATCACCTCTTGAACACCTT |
| AT1G60410 | FW | CACATTTCACGAACTGACCATCTC |
| | RV | AACGAAGCATCCAAGCCAAA |
| AT3G11310 | FW | GGGAAAACTACTCCAAGGAAAATAAGA |
| | RV | CTTTCCAGGTTCTACATGTCCTTTG |
| AT1G17770 | FW | TGGGATGTATCTGGTTTCAAAGTTC |
| | RV | AGCAGGAGGTTGGTTTGGTTT |
| AT2G36560 | FW | ACGATTCTACCTCACGGTTCAAG |
| | RV | CGCTCTCAGATATCGCCATTG |
| AT1G57800 (VIM5) | FW | GACGAGTAAAGGAAAGAAACCCATC |
| | RV | AGCATAAGAGCCAAAGCAAAGAGT |
| AT1G48910 (YUC10) | FW | AACCAACACTCAATCCCAAACG |
| | RV | TGGCTAAGTGAAGTTTGAGACGAT |

## (Table S6 continued)

Primers used for generating reporter lines

| gene | | Sequence 5' – 3' |
|---|---|---|
| AT1G43580 | FW | AACCTTCAGATGACTAAAGGTGGACTTGGAATAGCC |
| | RV | CCCTTGCTCACCATTGATATCGTGCCGTCGCTTGTCAAAGT |
| AT1G47530 | FW | TTTTTTCACATGGGAAAGGATAAGACTTTGCCG |
| | RV | CCCTTGCTCACCATTGATATCAGCTCCTGCGCCGT |
| AT1G64660 | FW | TTAACTACTATGGCTCATTTCCTCGAGACACAG |
| | RV | CCCTTGCTCACCATTGATATCAGCATTCTGAGGAATGCTTTCTCG |
| AT2G30590 | FW | GGTTCTTAGATGGAGGAGATAGAAGGAACAAACAGAG |
| | RV | CCCTTGCTCACCATTGATATCAGAGTTGTTATAGCTTGAGATGGCAAT |
| AT4G15390 | FW | GAGAAATCCATGGAGACGATGACGATGAAGGT |
| | RV | CCCTTGCTCACCATTGATATCAGAACCAAGACACTAGGGTTCATGG |
| AT5G53160 | FW | AGGAAGAAGATGGAAGCTAACGGGATTGAGAAC |
| | RV | CCCTTGCTCACCATTGATATCAGGACTCTCGATTCTGTCGTGT |
| AT2G33620 | FW | CCCTTGCTCACCATTGATATCCTTCCAGGGCATGTTAATGGTGTTATTG |
| | RV | GAGAAGAAAATGTCAGGATCTGAGACGGGT |

| Promoter | | Sequence 5' – 3' |
|---|---|---|
| AT1G43580 | FW | AAGCTAAGCTCTAGTGATATCCCATATATGTTCAACCAAATCAGCTTTTAAGTGT |
| | RV | TTTAGTCATCTGAAGGTTGATGGGTCGCC |
| AT1G47530 | FW | AAGCTAAGCTCTAGTGATATCGTGGGAAATTTATTTTGGAATATTTTAGGCGAAAAAATA TTAATATTTCA |
| | RV | CTTTCCCATGTGAAAAAAGCTCTGTTTTAGTTTCTCTTCTTT |
| AT1G64660 | FW | AAGCTAAGCTCTAGTGATATCAGAAATGTTGTTACAATCTTGTATTTGTTTGACTATATAT ATGAATGA |
| | RV | ATGAGCCATAGTAGTTAACTAAAGAAAAAGTAGAGCCTTTGTTAGGA |
| AT2G30590 | FW | AAGCTAAGCTCTAGTGATATCTAGTATTGGAATCGCTGTGGTGTTTGT |
| | RV | CTCCTCCATCTAAGAACCCTAATTTTTTCCCCCAGAAAAC |
| AT4G15390 | FW | AAGCTAAGCTCTAGTGATATCAAAACATAAAGAAAGATATTGTACAATTGTGGTAGGT |
| | RV | CGTCTCCATGGATTTCTCTCCTCTTTAGACGAAGAGAAG |
| AT5G53160 | FW | AAGCTAAGCTCTAGTGATATCGCTCTTCGAGTCAGGAAAAGTTAAGACT |
| | RV | AGCTTCCATCTTCTTCCTCCGTCTTAAGCCACG |
| AT2G33620 | FW | TCCTGACATTTTCTTCTCACTTTCCCAAACCAAAACAG |
| | RV | AAGCTAAGCTCTAGTGATATCATTAAGCCGGTTTTTGCGTGAC |

**Table S7.**

Quality of sequencing samples.

Table shows number of parental-specific reads in the samples analyzed in this study. Replicates are biological replicates.

| Replicates | Col reads | L*er* reads | Observed Ratio Col/L*er* | Expected Ratio Col/L*er* | Contamination |
|---|---|---|---|---|---|
| Col × L*er* rep2 | 1 532 326 | 589 982 | 2,60 | 2,0 | 9% |
| Col × L*er* rep3 | 3 527 140 | 1 155 593 | 3,05 | 2,0 | 14,9% |
| L*er* × Col rep2 | 1 157 086 | 2 048 590 | 0,56 | 0,5 | 0% |
| L*er* × Col rep3 | 1 566 319 | 3 177 167 | 0,49 | 0,5 | 0,30% |
